# Assessing the recovery of Y chromosome microsatellites with population genomic data using *Papio* and *Theropithecus* genomes

**DOI:** 10.1038/s41598-023-40931-x

**Published:** 2023-08-24

**Authors:** Giacomo Mutti, Gonzalo Oteo-Garcia, Matteo Caldon, Maria Joana Ferreira da Silva, Tânia Minhós, Guy Cowlishaw, Dada Gottelli, Elise Huchard, Alecia Carter, Felipe I. Martinez, Alessandro Raveane, Cristian Capelli

**Affiliations:** 1 Department of Chemistry, Life Sciences and Environmental Sustainability, University of Parma, Parco Area Delle Scienze, 11/a, 43124 Parma, Italy; 2 Barcelona Supercomputing Centre (BSC-CNS), Plaça Eusebi Güell, 1-3, 08034 Barcelona, Spain; 3 Institute for Research in Biomedicine (IRB Barcelona), The Barcelona Institute of Science and Technology, Baldiri Reixac, 10, 08028 Barcelona, Spain; 4 BIOPOLIS Program in Genomics, Biodiversity and Land Planning, CIBIO, Campus de Vairão, Vairão, Portugal; 5 Centro de Investigação Em Biodiversidade E Recursos Genéticos, CIBIOInBIO Laboratório AssociadoUniversidade Do Porto, Campus de Vairão, Vairão, Portugal; 6 ONE ‑ Organisms and Environment Group, School of Biosciences, Cardiff University, Sir Martin Evans Building, Cardiff, UK; 7 Centre for Research in Anthropology (CRIA-NOVA FCSH), Av. Forças Armadas, Edifício ISCTE, Sala 2w2, 1649-026 Lisboa, Portugal; 8 Anthropology Department, School of Social Sciences and Humanities, Universidade Nova de Lisboa (NOVA FCSH), Av. de Berna, 26-C, 1069-061 Lisboa, Portugal; 9 Institute of Zoology, Zoological Society of London, Regent’s Park, London, NW1 4RY UK; 10 Institut Des Sciences de L’Evolution, CNRS, Universite de Montpellier, CC 065, 34095 Montpellier 05, France; 11 Department of Anthropology, University College London, 14 Taviton Street, London, WC1H 0BW UK; 12 Escuela de Antropología, Facultad de Ciencias Sociales, Pontificia Universidad Católica de Chile, Santiago, Chile; 13 Human Technopole, Viale Rita Levi-Montalcini 1, 20157 Milan, Italy; 14 Department of Biology, University of Oxford, 11a Mansfield Road, Oxford, OX1 3SZ UK

**Keywords:** Biological techniques, Computational biology and bioinformatics, Evolution

## Abstract

Y chromosome markers can shed light on male-specific population dynamics but for many species no such markers have been discovered and are available yet, despite the potential for recovering Y-linked loci from available genome sequences. Here, we investigated how effective available bioinformatic tools are in recovering informative Y chromosome microsatellites from whole genome sequence data. In order to do so, we initially explored a large dataset of whole genome sequences comprising individuals at various coverages belonging to different species of baboons (genus: *Papio*) using Y chromosome references belonging to the same genus and more distantly related species (*Macaca mulatta*). We then further tested this approach by recovering Y-STRs from available *Theropithecus gelada* genomes using *Papio* and *Macaca* Y chromosome as reference sequences. Identified loci were validated in silico by a) comparing within-species relationships of Y chromosome lineages and b) genotyping male individuals in available pedigrees. Each STR was selected not to extend in its variable region beyond 100 base pairs, so that loci can be developed for PCR-based genotyping of non-invasive DNA samples. In addition to assembling a first set of *Papio* and *Theropithecus* Y-specific microsatellite markers, we released TYpeSTeR, an easy-to-use script to identify and genotype Y chromosome STRs using population genomic data which can be modulated according to available male reference genomes and genomic data, making it widely applicable across taxa.

## Introduction

In recent years, the development of new sequencing technologies has provided researchers with a flood of data whose characterisation has been made possible by the improvement of computational capacity and the implementation of novel tools. Given the significant insights that can be gathered, the analysis of whole genome sequences usually focuses on autosomal variation, and several tools have been developed to analyze such datasets^1,2^. However, data from other genetic systems, namely mitochondrial DNA (mtDNA) and Y chromosome, can be also retrieved from these datasets. Several pipelines have been developed for assembling mitogenomes from whole sequence data, many designed to control for possible biases related to the choice of the selected reference sequence^3–5^. Differently, Y chromosome sequences have been often overlooked, despite the utility of this system in shedding light on sex-related population dynamics, such as dispersal, mating strategies and reproductive success^6–11^. Humans are one of the few species where bioinformatic tools designed specifically for handling Y chromosome data have been assembled, with a focus on SNP variation and phylogenetic assignment^12–16^. However, Y-SNP data, while informative when combined with detailed phylogenetic information, can be of limited use when attempting to disentangle small scale dynamics, in case of related lineages, or when the underlying phylogeny is unknown, completely or in some of its parts. The biallelic status of most Single Nucleotide Polymorphism (SNPs) also makes them less suitable for an effective characterization of variation when the amount of available DNA is small. In such cases, the use of more variable markers such as Short Tandem Repeats (STRs) can provide higher discrimination power. These genetic systems can be also easily multiplexed to co-genotype several markers together, using PCR approaches that combine different amplicon sizes and the use of different fluorescent labels^17,18^. The use of PCR-based analyses as well as the possibility of selecting amplicons of different sizes have made STR the genetic markers most commonly used to investigate population dynamics in the wild, as for example reconstructing the degree of kinship between members of groups, by permitting the genotyping of non-invasive samples as hair and feces^19–21^.

The development of novel STR markers has been for a long time a tedious and laboratory intense activity. The procedure usually started with the screening of genomic DNA for the occurrence of regions with repeated motifs, then followed by the investigation of their degree of variation via the analysis of these regions across several individuals^22,23^. The availability of whole genome sequence data allows performing the same steps in silico, taking advantage of the genomic data often already available as part of genomic investigations. Software such as Tandem Repeat Finder (TRF) can be used in the first place to identify tandem repeats in an assembled reference sequence^24^. Then, other tools specifically developed to deal with this kind of data, such as HipSTR, can be used to accurately genotype each sample in the previously identified regions^2^. One of the most recent examples of the potential for similar approaches has been the development of an extended panel of STRs valid across catarrhines developed by identifying in silico homologies between human STRs and available catarrhines genomes^25^.

Following these considerations, we propose a bioinformatic program which we named TYpeSTeR to identify Y chromosome microsatellites integrating these tools and we assess its performance using genomic data with varying levels of coverage of individuals belonging to the genera *Papio* and *Theropithecus.* Beside assembling the first panel of *Papio* and *Theropithecus* Y chromosome microsatellites, our results offer a proof-of-concept for efficiently using genomic population data to recover and genotype Y chromosome microsatellites across taxa.

## Results

### Identification of repeated regions in the reference Y chromosome sequence

**Figure 1:**
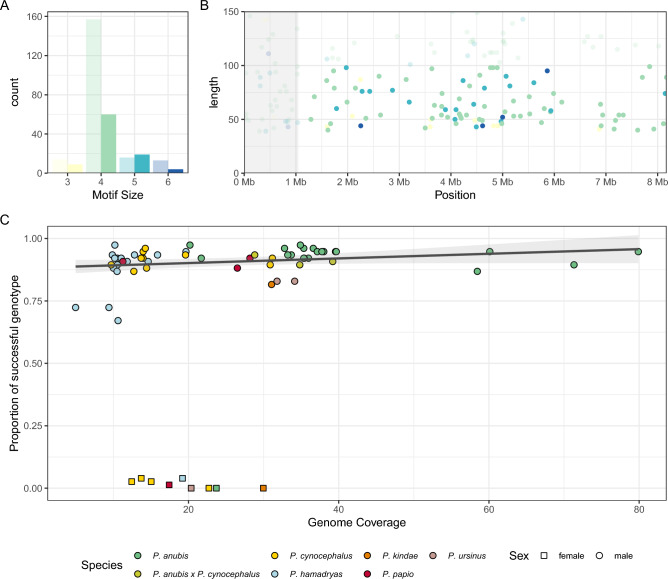
(**A**) Distribution of tandem repeat motif sizes. Shaded columns refer to unused sites that either fell in the Pseudo Autosomal Region (shaded in gray in panel (**B**)) or exceeded the maximum length (100 bp; panel (**B**)) (**B**) Chromosomal location and maximum length of identified loci. Colours as in panel (**A**). (**C**) Proportion of successful genotypes against whole genome sequencing coverage of each sample, coloured by species and shaped by sex.

A total of 292 repeated regions (excluding dinucleotides and repeats longer than 6 bp) were identified across the *Papio* Y chromosome reference (NC_044997.1). Ninety-two were found in the pseudoautosomal region and were excluded from further analysis. Overall, the most represented motifs were tetranucleotides (Fig. 1A). Using our selection criteria (see "Materials and Methods"), a total of 92 loci were finally selected. The localisation and the length of the loci across the reference sequence is shown in Fig. 1B.

### Using HipSTR for genotyping genomic data

**Figure 2:**
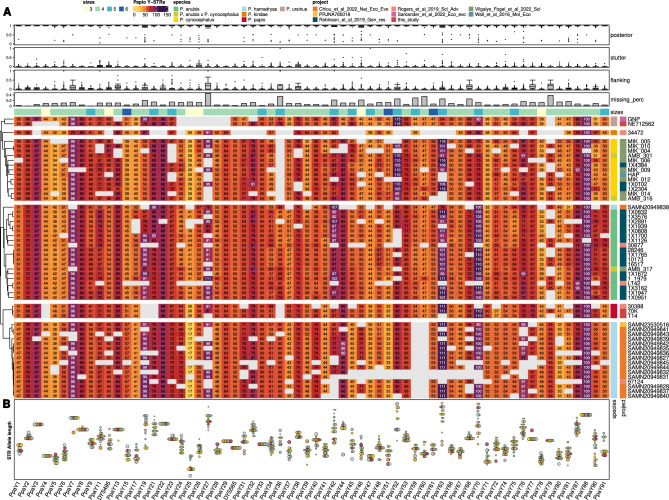
(**A**) Heatmap coloured by the length of each allele in the *Papio screening* dataset. Samples are clustered according to the phylogeny as described in "Materials and Methods". Sites (columns) are annotated with boxplots of (i) genotype quality, (ii) proportion of reads with a putative stutter artifact, (iii) proportion of reads with an indel in the flanking region, (iv) bar plot number of missing data and finally (v) coloured bar of motif sizes as coulor-coded in legend. Each sample (row) is annotated with a coloured bar of (i) species and (ii) project. (**B**) Summary of the heatmap in panel A with the distribution of allele length. Samples are divided by alleles and species, each dot size is scaled by the relative proportion in each species (e.g. if two out of three *P. papio* samples share the same allele the dot will be 2/3 of the maximum size). Sites are identified using the nomenclature proposed in "Materials and Methods". DYS495 and DYS565 are markers homologous to human Y-STRs and the human label has been therefore indicated here. The heatmap was produced with ComplexHeatmap v.2.8.0 R package^56^.

We proceeded by genotyping the selected 92 STRs in the panel of 55 baboon individuals sampled from all the six *Papio* species (see "Materials and Methods") using the software HipSTR^2^. Ten of the putative regions were not genotyped as virtually no reads were mapped to these sites, while six additional sites were automatically filtered out by HipSTR as the upstream or downstream flanking regions were too repetitive, leaving a total of 76 loci genotyped. Among these, only sites with < 50% missing data and no reads from female samples mapped were further retained, leading to a final set of 66 loci. Notably, only three STRs were mapped to female individuals with more than two reads, irrespectively of the coverage of the samples, providing strong support for the male specificity of marker identification and genotyping procedures. These three sites were therefore removed in all analyses. Across the 66 putative Y chromosome STR markers, the number of successfully genotyped microsatellites varied across individuals and correlated with the degree of coverage (Fig. 1C). STR based Y chromosome haplotypes were then generated by assembling the alleles called at each locus by HipSTR (Tab S3AB; Fig. 2).

Genotyping failures were mainly due to two reasons: sequencing issues and genomic features of the sample. The genotype posterior probability was mainly driven by the quality of the sequencing process. Samples with lower Phred quality score (on average), such as the only *P. kindae* (sample 34472) from Ref.^26^, had also lower mean posterior probability values. Moreover, one *P. anubis* sample from Ref.^27^, sample 1X2891, despite being high coverage (32.8X) had a peculiar issue in the reads as the first couple of bases had very high percentages of missing data. Indeed, this sample had a lower mean posterior quality (mean = 0.926 ± 0.212) compared to all other samples from the same project (mean = 0.996 ± 0.05) (Fig. S2A). Overall, in order to genotype most of the STR loci, a sample had to be at least ~ 10X in coverage, although one *P. hamadryas* sample with lower coverage (5X) had still quite a high rate of successful genotypes (72.4%) (see Fig. 1C).

We also noted that individuals of the same species were consistent in showing missing data at the same loci (Fig. 2A; Tab. S3AB), which we suspected might be due to the presence of structural variation across *Papio* Y chromosome lineages. We therefore explored the degree of coverage along the Y chromosome reference across the 55 *Papio* genomes in the screening panel (Fig. S3). For example, we identified a potential *P. ursinus*-specific deletion spanning six markers from 3.5 Mb to 3.8 Mb which indeed HipSTR fails to genotype in both the *P. ursinus* samples in our dataset as no reads were mapped there. Similarly, two out of 16 *P. hamadryas* samples possibly shared a large deletion from 4.4 Mb to 5.7 Mb and none of the 15 STRs included were genotyped in these samples. The *P. ursinus* specific deletion was confirmed by *P. ursinus* unpublished long-read genomic data (not shown).

The number of invariant sites per species ranged from 21 in *P. anubis* to 57 in *P. papio*. Sixteen out of the 66 sites were monomorphic within at least one of the two *North–South Papio* clades. Eight of these loci were invariable across all samples. On the opposite side, other eight sites were extremely variable (more than seven different alleles). Of the three more represented species, *P. anubis* had a median number of four alleles per site (mean = 3.59 ± 1.90), *P. cynocephalus* of three (mean = 3.12 ± 1.70) and *P. hamadryas* of two (mean = 2.20 ± 0.95) (Fig. 2C).

Y-STR diversity (see "Materials and Methods"; Nei, 1973) in the *Papio sp.* dataset was 0.584. *P. hamadryas* baboons showed the lowest heterozygosity (0.126) compared to the other two most represented lineages: *P. anubis* (0.439) and *P. cynocephalus* (0.370). Indeed, some degree of further clustering seems to be present in all these three species of *Papio*. *P. anubis*, in particular, appears to display two main, well-represented and highly differentiated clusters (samples are all from Kenya), while *P. cynocephalus* cluster contains at least two highly divergent lineages represented by one and two individuals respectively, all collected in Mikumi National Park, Tanzania.

### Validation of genotyped Y-STRs

We used the Y STR genotypes to build a phylogenetic tree of the different haplotypes. Haplotypes of individuals belonging to the same species clustered together, with no evidence of polyphyletic status and in line with known *Papio* phylogeny, subdividing Northern and Southern baboon species^26^. The Y chromosome data is further supporting the suggestion that sample 1X3576 was mislabeled as *P. cynocephalus,* instead of being assigned to *P. anubis*^27^. We also identified in 1X4384, reported as a *P. anubis* founder but genetically closer to *P. cynocephalus*^27^, a Y chromosome haplotype that clustered with other yellow baboons. The Y chromosome haplotype of one *P. hamadryas* sample (SAMN20949838) clustered with olive baboon lineages, indicating a possible recent hybridization event. Two out of the three high coverage hybrid samples from Amboseli had a Y-STRs haplotype more similar to other yellow baboons (AMB_316 and AMB_301), while the remaining one (AMB_317) was more similar to olive baboons. In these six cases, we assigned the species of the sample based on these clustering results.

Four of the genotyped samples in the *Papio Screening* dataset were related across three generations along their paternal line^28^. The four individuals belonged to the species *Papio anubis* and have been sequenced at high coverage (mean coverage = 28X). The four Y-STRs haplotypes were fully concordant as expected, as each allele at any given locus was the same in all four individuals (Fig. 2; Tab. S3AB). Furthermore, two different sequencing runs (97124 and SAMN20949845; peer reviewer personal communication) of the same *P. hamadryas* sample returned fully concordant haplotypes.

**Figure 3:**
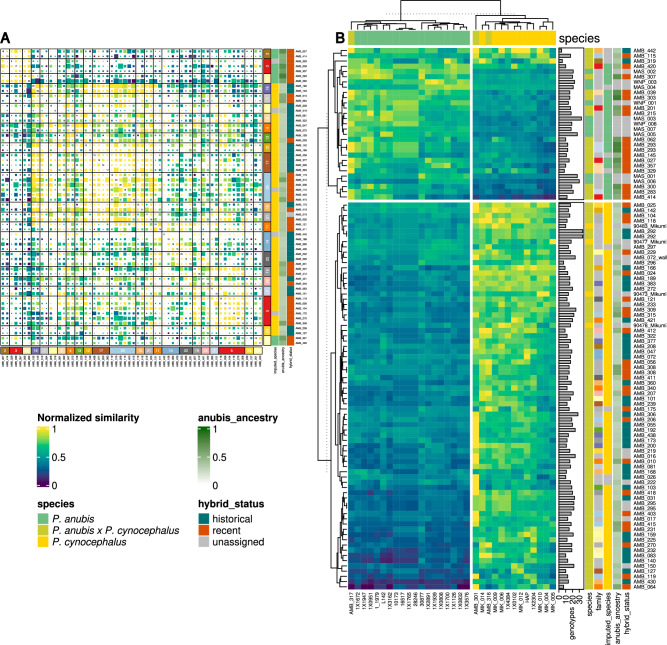
(**A**) Pairwise normalized distance (number of equal alleles divided by total number of shared alleles) between pedigreed samples from *Low coverage* dataset. Samples are grouped by pedigree (Fig. S4) and groups are clustered according to the heatmap values. The size of each panel is proportional to the number of shared alleles (i.e. the denominator of the pairwise normalized distance). The coloured bars represent a numerical id for each family, the imputed species (as described in main text), the *P. anubis* ancestry and the hybrid status (both as reported in ^29^). (**B**) Pairwise normalized distance between high coverage *P. anubis* and *P. cynocephalus* samples (columns) and low coverage samples (rows). Both rows and columns are clustered by k-means. The barplot represents the number of genotypes for each low coverage sample. Coloured cells give the information to: the species, the family (as coloured as in panel A), the imputed species and the *P. anubis* ancestry and hybrid status (as reported in Ref.^29^). Both heatmaps were produced with ComplexHeatmap R package^56^.

Reassured by this result, we also analyzed the recently published low coverage sequence data of hybrid baboons from Amboseli National Park included in the *Papio LowCov* dataset^29,30^, and recovered Y-STRs haplotypes for most of the available male individuals (n = 225). We used publicly available trios to reconstruct pedigrees among individuals and combined this information with the recovered Y-STRs haplotypes (Fig. S4; "Materials and Methods"). As expected, the number of successfully genotyped Y-STRs was substantially affected by the extent of coverage of the analyzed genomes (Fig. S1A). However, in the range of genome coverage of this dataset (0.6X to 3.5X), the number of successfully genotyped loci rises linearly with coverage. Interestingly, samples with a genome coverage of at least 3X have more than 75% of the STR loci being genotyped, reaching the same degree of genotyping success observed for higher coverage samples (Fig. 1C and Fig. S1A). The number of STRs genotyped and shared among pairs of individuals within each pedigree ranged between 0 and 10 (median = 4), while the range observed across pedigrees was larger (range 0–21, median = 4). Within pedigrees, seven occurrences of allele mismatch were observed out of 282 total possible comparisons (2.5%): two of these occurred in the same locus. Two families had two mismatches each (Fig. 3A, family 13 and 25). Within-pedigree haplotypes were usually closer to each other than to other haplotypes, confirming the presence of related male lineages. Interestingly, we found a few instances of strong similarity across pedigrees: for example pedigrees 19, 26, 7 and 5 or 12, 17 and 25, hinting to deeper relationships along the male lineage for these individuals (Fig. 3A).

Haplotypes recovered from the *Papio LowCov* dataset were finally compared to haplotypes retrieved from high coverage genomes of *P. cynocephalus* and *P. anubis* present in the *Papio Screening* dataset (Tab. S3GH). Notably, despite the small number of genotyped STRs in the *Papio LowCov* dataset, a substantial fraction of haplotypes recovered from the latter dataset was significantly closer to either yellow (68%) or olive baboons (22%) (Fig. 3B). Only one of the 14 samples from the non-hybrid areas investigated in Ref.^30^ (sample MAS_004; an olive baboon from the Maasai Mara Reserve in Kenya) was not assigned to any of the two species. Interestingly, all the Amboseli hybrids with a *P. anubis* Y-STR haplotype have been previously classified as recent hybrids (defined as having at least one *P. anubis*-like ancestor within the last seven generations, as described in Ref.^29^).

### STR identification using Y chromosome references of other genera

In order to explore the potential for STR identification using increasingly evolutionary distant Y chromosome reference sequences, we extended our approach to population genomic data of two different genera (from the *Papio Screening* dataset and *Theropithecus* dataset) using less closely related references (*Macaca mulatta* and *Papio anubi*s). More precisely, *Papio* and *Theropithecus* divergence is dated ~ 4.5 Million years ago (MYA) (CI 4.0–5.1 MYA) and both diverged from *Macaca* ~ 10.5 MYA (CI 8.8–11.1 MYA)^31^. This test allowed us to assess the performance of the proposed workflow when only references from related species are available.

**Figure 4:**
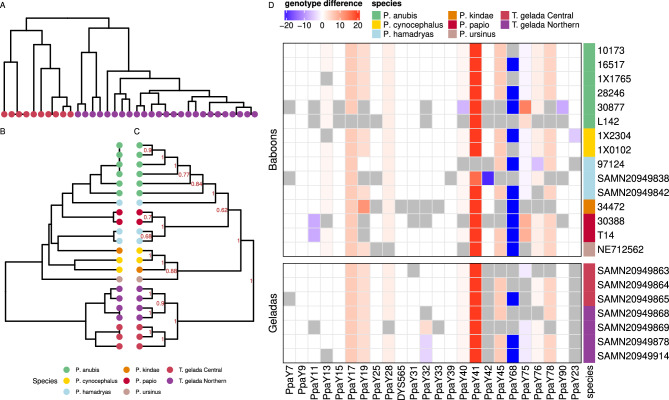
(**A**) Phylogenetic relationships among *Theropithecus gelada* Y chromosome haplotypes generated using *Papio*-discovered STRs. Colours refer to demes of origin (see legend). (**B**) Phylogenetic relationships among Y chromosome haplotypes generated from a selection of *Papio* and *Theropithecus* individuals (*Macaca* dataset) using *Macaca*-discovered STRs. Colours refers to the demes/species of origin as described in the legend. (**C**) Phylogenetic relationships among Y chromosome haplotypes of individuals in the *Macaca* dataset generated by resampling *Papio*-discovered STRs, as described in the main text. Colours refers to the populations/species of origin as described in the legend. The proportion of times where the node is observed when resampling STR markers is reported in red. (**D**) Difference in allele calling over loci shared across *Papio*-based and *Macaca*-based STRs. Colours in the Species column refer to legend in B/C. The heatmap was produced with ComplexHeatmap v.2.8.0 R package^56^.

Indeed, as no Y chromosome reference sequence is available for *Theropithecus gelada*, we used the information retrieved by TRF on the *Papio* Y chromosome reference sequence and applied HipSTR to genotype the set of 92 initially selected loci across the full *Theropithecus* dataset. Of these 92 sites, 20 loci were dropped due to the lack of reads mapping to the corresponding regions and seven were filtered due to the high repetitiveness of the flanking sequences. Fourty-nine out of these 65 loci were finally retained after genotyping once the quality control filters used above were applied (see "Materials and Methods"; Fig. S2B, Tab. S3CD). In this case, only one site was filtered out as it was also genotyped in females. We note that of the starting 92 loci identified in the *Papio* reference, more were retained when tested in *Papio* than *Theropithecus* genomes, but over half were still usable in *T. gelada* (66/92; 72% and 49/92, 53%, respectively). Across the two sets of loci the vast majority, 48, were shared (PpaY62 was the only marker only found in *T. gelada*). We used the 49 markers to build a phylogenetic tree, as done for the *Papio screening* dataset. *T. gelada* Y-STRs haplotypes clustered in two groups, corresponding to two demes of origin (Northern and Central), zoo samples clustered with the Central population, as per their genome^32^ (Fig. 4A). Twenty-five out of the 49 (51%) sites were polymorphic. The Y-STR diversity^33^ within the two populations of *T. gelada* was 0.213 and 0.148, respectively, in the central (including zoo samples) and northern populations and 0.213 overall.

The *Macaca* Y chromosome reference was initially processed with TRF and a total of 342 variable regions were identified using the same criteria applied for the identification of Y STR on the *Papio* Y chromosome reference. One-hundred and forty-four of these regions passed all screening filters. Of these, 89 were considered by HipSTR, and 61 were finally retained after genotyping in a subset of 15 *Papio* samples, and seven *Theropithecus* samples once all the genotyping filters applied before were taken into consideration (see "Materials and Methods").

To explore how the use of references at different evolutionary distances could affect the identification and genotyping of potential Y-STRs, we focused on the same set of 15 *Papio* and seven *Theropithecus* individuals as described above. We then estimated how many STRs were retained after filtering and how many were variable when *Papio*-discovered and *Macaca*-discovered STRs were considered. The fraction retained is 63/145 (43%) when using *Macaca* compared to 66/92 (71%) when using *Papio* as reference. The fraction of variable sites in the *Macaca* set is smaller than that of the *Papio* set (69% and 86%, respectively, for *Papio* genomes; 35% and 45%, respectively, for *Theropithecus* genomes). Notably, alleles in loci identified in *Macaca* are consistent across a set of *Papio* individuals related along the paternal line across three generations, confirming consistency in the genotyping (Tab. S3E). Also, the retrieved *Macaca*-based STRs separate the *Papio* genomes in groups reflecting different species, and *T. gelada* samples in corresponding populations but fail to recover the Northern-Southern *Papio* subdivision identified by the *Papio*-discovered markers (Fig. 4B). It is worth noting here that in the reconstruction of the baboon phylogeny the set of available variable STRs was ~ 30% larger for loci discovered in *Papio* than in *Macaca* (57 vs 44 for *Papio* and 25 vs 19 for *Theropithecus*), which might explain the observed differences in how main clusters are related (Fig. 4BC). We indeed generated a phylogenetic tree of the same set of 22 *Papio*/*Theropithecus* individuals and tested the support for the retrieved topology by resampling the 44 of the 66 *Papio*-discovered loci 100 times (Fig. 4C). Most of the nodes are strongly supported, including the monophyletic status of all the Southern baboons but not the grouping of all of the Northern baboons, suggesting how different sets of markers, as expected, provide different degrees of support to reported phylogenetic signals. Interestingly, when STRs identified as homologous in *Papio* and *Macaca* Y chromosome references (24 markers) are compared in their genotypes, a good degree of consistency in allele calling is observed. Discrepancies are noted mostly in samples that were poorly genotyped (Fig. 4D). Some of the loci, however, showed discrepancies consistent across all samples. This could be related to loci being misclassified as homologous between *Papio* and *Macaca* and/or the genotyping by HipSTR being reference biased. We manually curated the alignments of the sequences of the genotypes called by HipSTR of the mismatched alleles reported in Fig. 4D to further explore this observation (Fig. S6). Differences present in some but not all individuals appear as true genotyping errors, possibly caused by the quality of the genotyped genomes (for example for PpaY11-23–32-42–90; see "Materials and Methods" for nomenclature). On the contrary, in the loci consistently showing a mismatch of the same degree in all the individuals (PpaY13-17-19-28-40-41-45-75-76-78) HipSTR appears to include a few extra nucleotides of the flanking sequence when samples are mapped to one or the other reference, genotyping an allele of different size (Fig. S6). Such discrepancies can be easily solved by aligning the genotyping results obtained using different references. In one case (PpaY68), the two homologous markers refer to two different but contiguous tandem repeats (with CCTC motif in *Papio* and CTCT in *Macaca*). HipSTR genotypes the first even when given the second as input when using *Macaca* as reference.

### Markers homology: intraspecies and human Y chromosome reference

Across all the identified loci, three markers were homologous to human STRs^12^. Only one of these sites (DYS565) was shared among all three reference species (*Papio anubis*, *Macaca mulatta* and *Homo sapiens*). A marker homologous to human DYS495 was found in *Papio,* whereas one homologous to human DYS391 was found in *Macaca*. The differential retention of homologous loci is probably related to lineage-specific Y chromosome rearrangements^6,34,35^. In these three cases, we identified the loci using the corresponding human label (Tab. S2, Fig. S5B). This degree of homology (3 out of 92 human markers) is in line with previous observations reporting 18 out of 136 human single-copy Y-STRs successfully amplified in mandrill^36^. We searched for the presence of multi-copy markers within each STR dataset by using the same criteria of identity and query coverage applied for the identification of cross-reference homologous loci. To validate this approach we screened the human Y STR panel and correctly recovered all except one (YCAIIa/b) of the 10 human multi-copy markers. In *Papio,* we identified 4 multi-copy markers (PpaY16 and 18, PpaY54-55–57, PpaY56-58 and PpaY84-85), while eight were found in *Macaca*. Finally, among the 43 homologous markers found between *Papio* and *Macaca,* two are multi-copy in baboon but not in macaque and three are multicopy in the latter but not the former (Tab. S2).

We also note that with our approach, the only Y chromosome STR previously genotyped and variable in *Papio,* the human homologous DYS576^37,38^, was filtered upstream as the locus is longer than 100 bp (NC_044997.1: 1681190–1681344). We nevertheless tried to genotype it in the *Papio* screening samples but HipSTR was not successful as no reads were found spanning across the repetitive sequence.

### In silico PCR evaluation of identified Y-STRs

We evaluated the feasibility of genotyping the selected loci using a molecular genetics approach by exploring the sequencing features of the regions surrounding the Y-STRs. Considering the ampliconic structure of extended parts of primates Y chromosome^39^, successfully designing PCR primers is not trivial. Therefore, we analyzed its feasibility with an in-silico approach taking as template the successful primers used in humans. Of the 92 *Papio/Theropithecus* loci, 74 were amenable to generate a pair of primers ("Materials and Methods"; Supplementary Fig. S7A). Of these 74, seven (~ 10%) were predicted to generate locus-specific PCR products. However, this number rises to 44 (~ 60%) when including PCR primer pairs predicted to amplify non-specific amplicons longer than 10 kb. Large amplicons are expected to be disadvantaged when competing with the amplification of substantially shorter regions (100–200 bp) and therefore their theoretical co-amplification can be considered not problematic in primer design (Tab. S4). For comparison, we explored in the same way the “*named*” set of 92 human Y-STRs^12^. Several of the Y chromosome human loci were surrounded by repeated regions, as for the non-human loci here identified (Fig. S7). Moreover, the two conserved Y-STRs between *Papio* and *Homo* (DYS495 and DYS565) show similar flanking repeats further validating their homology.

We then focused on 17 primer pairs amplifying a set of 20 human Y STR loci routinely investigated in forensic genetic analysis^40^ and tested the specificity of the available PCR primers. The primers for DYS388 were not found in hg38 therefore we excluded this locus. Of the remaining 16 sets of primers 10 (63%) were locus-specific and the other six were predicted to co-amplify non-specific genomic regions larger than 10 kb (Fig. S7B). It is worth noticing here that each human primer set was manually curated when originally designed and further refined after initial molecular testing.

### TYpeSTeR pipeline

We assembled the approach used here for retrieving and genotyping Y-STRs in a simple-to-use script we named TYperSTeR, which is available at the dedicated github page (https://github.com/giacomomutti/TYpeSTeR). This program makes it possible to identify and genotype a panel of Y-STRs in the species of interest, simply using mapped sequencing samples (for example from a genome resequencing project) and a Y chromosome reference (or W in birds and other species with a similar sex-determination system). The user can specify maximum motif length, maximum STR length and which regions to exclude in the analysis. Further, we included a script to assess the homology between Y-STRs of different Y chromosome references. The final outputs are the putative Y-STR regions and the genotypes in these regions for each sample as a Variant Call Format (VCF) file. This file can be further analyzed with supplementary scripts included in the repository. The dependencies of the tool are easy to install and it may also be used as a Singularity container.

## Discussion

The explosion in the amount of available genomic data has made the application of genome-based approaches for the identification and characterisation of genome-wide markers feasible in species besides humans. However, despite a decrease in the sequencing costs, population-based genomic analyses are still often prohibitive, and cost-effective genotyping approaches are still sought, the choice of markers depending on the aims of the investigation. Here we propose an approach for the discovery and genotyping of Y chromosome STRs that can be used in different contexts. Using genomic data to search for variable loci, we identified a similar number of potentially variable STR markers in *Papio* and *Macaca*, 292 and 342, respectively. Once filtered for repeat size, number of repeats, localisations (outside PARs), and variability in the same set of individuals, the fraction of retained markers was larger for the *Papio*-discovered set than the *Macaca*-discovered set (87% and 68%, respectively). This trend was also observed in *Theropithecus* (45% and 33%), suggesting, as expected, that as evolutionary distance increases, the number of successfully retained and genotyped markers decreases. However, the results in both *Theropithecus* and *Papio* clearly indicate that the lack of a closely related Y chromosome reference does not prevent the identification of a set of usable and informative markers, able to recover relationships among Y chromosome lineages. Alleles called in loci identified in *Macaca* are consistent across male-related individuals and reproduce the main phylogenetic signals associated with *Papio*-discovered loci, both in *Papio* and *Theropithecus* (Fig. 4B–D). No mismatches in species/demes assignment were noted in both *Papio* and *Theropithecus* when comparing the clustering results using *Papio*-based and *Macaca*-based markers (Fig. 4B,C). Variation in the relationships between clusters appears mostly shaped by the set of analyzed markers (Fig. 4B,C). The choice of the reference does not affect the degree of genotyping success; the latter is mostly affected by the quality of the analysed genome. More specifically, lower genotyping success is associated with lower genome quality. Differences in genotyping across the same samples/same loci using different references are mostly related to the quality of genomic data used and not by what reference was used (Fig. 4D). Notably, a coverage as low as 3X provides still a good genotyping score, which might suggest this could be used as a sequencing threshold for confidently recovering such type of data. Low coverage data provided interesting insights on the affinity among male pedigrees and identified the species of origin of the Y chromosome of hybrids, showing the potential for markers discovered with the approach presented here. Specifically for the species analysed, we note that besides the identification of the main clusters (main *Papio* species, different populations in *T. gelada*), further topological features within the reported phylogenies should be treated with caution as STRs are less reliable than SNPs in identifying relationships among clusters^41^. In addition, both the *Papio Screening* and *Theropithecus* datasets do not provide an exhaustive sampling of the variation present in the related species and therefore, the reported topology cannot be considered fully representative of the phylogenetic relationships.

The successful identification of informative markers, even when using evolutionary distant reference Y chromosome sequences, suggests that this approach could be applied more extensively. This is particularly of relevance considering that a total of 30 Y chromosome references and WGS from 86% of primate genera became available following recent publications^39,42^. Targeting species in key primate groups to assemble Y chromosome references would make possible a more extensive application of the approach presented here. For example, Strepsirrhini are still underrepresented in genomic data and should be a priority given the lower density of reference male genomes within this part of the primate phylogeny. More generally, we note that Y chromosome references are available also for other non-primate mammals, making the approach presented here potentially transferable to other mammalian orders and possibly even extended to W chromosomes in birds. We suggest the STRs identified using TYpeSTeR could be a useful by-product of resequencing projects, where many samples are mapped to the same reference, even at low coverage (~ 5X) and could link a purely in silico analysis to experimental work. Indeed, the markers identified using this approach could be genotyped via PCR once appropriate primers are developed. As a proof of concept for using TypeSTeR to identify Y chromosome STRs compatible with molecular genotyping we validated in silico PCR primer sets for 44 of the 92 loci. More could be possibly designed by focusing specifically on selected loci. Nevertheless, we envisage that researchers might prefer to develop their own set of primers according to their specific needs, particularly when building multiplex PCR protocols. Notably, markers selected using TYpeSTeR could be easily genotyped also in samples characterised by degraded and/or low amount of endogenous DNA, retrieved from non-invasive material and ancient specimens. The analysis of these samples could be either completed by assessing low coverage genomes or by PCR, as the parameters we tested make the genotyping feasible with both approaches.

## Materials and methods

### Whole genome population datasets

We assembled a whole genome sequence screening dataset consisting of both available and unpublished 55 *Papio* high and low coverage genome sequences (mean coverage = 26.2X ± 16.2X, Tab. S1). All of the six *Papio* species were included, but different numbers of individuals per species were available (range 1–18; Tab. S1). The final dataset included one *Papio kindae,* two *P. ursinus*, three *P. papio*, three *P. anubis x P. cynocephalus* hybrids, 11 *P. cynocephalus,* 17 *P. hamadryas* and 18 *P. anubis* (four of the latter related along the paternal line: grandfather, father and two sons). The Y chromosome data of one *P. ursinus* (sample NE712562) and two *P. papio* (T14 and 70K) are presented here for the first time. We additionally included 9 *Papio* female genomes to validate the male-specificity of the recovered markers (Tab. S1). This dataset, referred to as *Papio Screening* dataset hereafter, was used to assess the potential of discovering new Y-STRs in animal populations from different, but closely related, species.

Further, we assembled another dataset comprising of 199 male samples from the recently published low coverage *Papio* genomes from Amboseli (mean coverage = 1.04X ± 0.19)^29^, 26 male samples from Ref.^30^ and the 32 *P. anubis* and *P. cynocephalus* samples from the *Papio Screening* dataset, for a total of 257 samples (*Papio LowCov* dataset). Pedigree data were publicly available for 77 out of 199 samples from Amboseli, allowing us to use this subset to assess the accuracy and informativeness of genotyping recovered from low coverage resequencing data (https://github.com/TaurVil/VilgalysFogel_Amboseli_admixture).

We also analyzed the *Theropithecus gelada* genome dataset released by Ref.^32^ which comprised a total of 68 genomes (35 males and 33 females), ranging in coverage between 1.3X and 47.5X (mean = 14.4X ± 10.7X, Tab. S1). Samples were from two of the three known *T.gelada* populations, the Northern and Central ones^43,44^. This will be referred to as the *Theropithecus* Dataset.

### Read mapping

Read mapping and alignment processing was performed as in Ref.^45^. The reads of all the baboon samples were mapped using bwa-mem2 v2.2.1^46^ to *P. anubis* (Panubis1.0^47^), whereas the *T. gelada* samples were mapped to a custom reference genome made of Tgel_1.0^32^ and the Panubis1.0 Y chromosome (NC_044997.1). Read mapping was performed with bwa-mem2 v2.2.1, if a sample had more sequencing runs, those were merged with samtools merge^48^. Duplicated reads were marked with Picard MarkDuplicates v.2.26.10^49^ and removed. Only properly paired reads with mapping quality greater than 10 were kept with samtools view -f 2 -q 10 v1.15.1.

### STR identification using same genus Y chromosome reference

We initially screened the Y chromosome sequence recovered from the Olive baboon (*P. anubis*) reference Panubis1.0^47^ for the occurrence of repeated regions using the TRF tool^24^. We then filtered these regions by using three criteria: size of the repeat unit being between 3 and 6 nucleotides, the presence of at least 8 repeats and a total length of the variable region no longer than 100 nucleotides. The first two parameters focus on markers easy to genotype using PCR analysis by limiting the extent of the impact of the so-called stutter peak (very pronounced when dealing with di-nucleotide STRs^40^. The minimum number of repeats is designed to maximize the chance of recovering interindividual variable markers. Similar parameters were applied when searching for Y chromosome human STRs^41^. We have additionally restrained the size of the overall variable region to 100 nucleotides because short reads can not be used to genotype long STRs, a feature that nevertheless makes the identified markers potentially amenable to characterisation in *less than optimum* DNA extracts, as the case for non-invasive DNA samples^50^.

Finally, sites in the Pseudo Autosomal Region (PAR) were excluded in order to filter out non haploid genotypes. The PAR was identified in the Y chromosome scaffold by aligning it to the human PARs (with coordinates 10,001–2,781,479 and 56,887,903–57,217,415 as reported in https://www.ensembl.org/info/genome/genebuild/human_PARS.html). In line with previous findings for Rhesus macaque (*M. mulatta*), we identified in *P. anubis* only a single PAR, from position 1 to 1,047,499, corresponding to human PAR1^35^.

### Y-STR genotyping and filtering

We used HipSTR v.0.6.2 to genotype our samples. HipSTR is a bioinformatic tool designed to genotype STRs from Illumina whole genome sequencing data by accounting for errors in the alignment which are caused by their repetitive nature and PCR stutter errors which in turn produce reads that may contain a different number of repeats compared to the true genotype^2^. For this analysis, HipSTR was used in haploid mode. Genotypes were considered as missing data when either more than 35% of reads had an indel in the flanking regions, had a stutter artifact or if the posterior was lower than 90%. Finally, sites with more than 50% of missing data were filtered out. Sites that had more than two reads mapped on the nine female samples were also excluded. The same parameters were applied to genotype *Papio* STRs in the *Theropithecus* dataset. For the *Papio LowCov* dataset, we used different parameters to account for the substantially lower average coverage: minimum posterior quality was set to 0.75 and the allowed proportion of missing data was raised to 0.75.

### Y-STR validation

In order to check the male specificity of the putative STRs, we included in our analysis nine *Papio* female samples, one each from the six species in Ref.^26^ and three *P. cynocephalus* samples from Ref.^29^. We similarly analyzed the 33 female *T. gelada* genomes in the *Theropithecus* dataset (Tab. S1). Sites with more than two reads mapped in these 42 samples were excluded. To validate the STR genotypes recovered using HipSTR, we used a phylogenetic and a genealogical approach. For the phylogenetic validation, we used the STR-based Y chromosome haplotypes to reconstruct the relationships among the samples. We estimated the genetic distance within samples with Bruvo distance^51^ as implemented in Poppr v. 2.9.3^52^. The resulting distance matrix was used to generate a phylogenetic tree with the Unweighted Pair Group Method with Arithmetic Mean (UPGMA) method. As a measure of Y-STR variation, we estimated Nei’s gene diversity (expected heterozygosity) for each locus, then averaged across loci^33^.

Genotyping accuracy of the inferred haplotypes was tested in two ways: by including samples with known pedigree and a sample sequenced in two different projects. Four samples were related along the paternal line over three generations (see Fig. 2B in Ref.^28^) and were sequenced in Ref.^27^ at high coverage; 77 samples were part of the *Papio LowCov* dataset and belonged to 23 families (21 with simple father-son relationships and two spanning three generations with six samples each^29^ (Fig. S4). Further, *P. hamadryas* sample 97124 from Ref.^26^ and sample SAMN20949845 from Ref.^32^ (annotated as BE97-124) are actually the same organism.

Given the high proportion of missing data in the *LowCov* dataset, we devised a normalized pairwise similarity index between samples computed as the ratio between sites with the same genotype and the number of sites genotyped in both samples. This index should therefore be 1 in samples sharing the same Y chromosome haplotype (as for example for male-related members of the same family, where pairs share the same alleles in all the loci in common) and 0 if two samples do not share any site with the same genotype. The value is NA if the set of loci recovered in the two samples do not overlap, each having its own combination of markers. The higher the index, the more similar the two compared haplotypes are. We used the same index to tentatively assign each of the 225 low coverage samples to either *P. cynocephalus* or *P. anubis.* We initially generated for each low coverage sample two distributions of this index by considering comparisons with the 13 *P. cynocephalus* and the 19 *P. anubis* high coverage samples, separately. We then compared for each sample these two distributions: If the adjusted p-value of a *wilcoxon-test* between the two distributions was less than 0.05, we assigned the sample to either *P. cynocephalus* or *P. anubi*s depending on the test statistics.

### Y-STR identification using evolutionary distant references

In order to explore the feasibility of identifying STR markers using more evolutionarily distant Y chromosome references, a subset of 22 samples (15 from the *Papio* screening dataset and 7 from the *Theropithecus* Dataset) were also mapped to *M. mulatta* (Mmul_10^35^). This will be hereby called the *Macaca* dataset. The same procedure and filtering steps were applied as done with the *P. anubis* reference.

### Homology detection between references and nomenclature

To assess which Y-STRs shared a common origin in the *Papio* and *Macaca* references, we performed an homology search with BLAST v.2.11.0^53^ for the sites that passed the selection criteria both in *P. anubis* and in *M. mulatta*, including an additional 200 base pairs (bp) to each of the flanking region. We also included the “*Named*” panel of Y-LineageTracker^12^ which contains all named loci (92 markers) found in humans. Two sites were considered homologous if the identity percentage was greater than 80% and the query coverage was greater than 65%. We used these criteria also to detect putative multi-copy STRs within the same reference.

If a marker was homologous to a human STR, we named it with the corresponding human label. We named the remaining STRs, combining the prefix “PpaY” followed by a progressive number starting from 1 according to their position along the reference chromosome. The markers shared between *Macaca* and *Papio* were therefore also named as “PpaY”. We labeled instead as “MmuY'' all the markers uniquely found using the *Macaca mulatta* Y chromosome reference sequence (NC_027914.1). A full list of the location of loci along the reference chromosomes and their proposed nomenclature is reported in Tab. S2.

### PCR primers design and evaluation

Repeated regions on the *P. anubis* Y chromosome were annotated with RepeatMasker v. 4.1.2-p1^54^ using the Primates repeats database. PCR primers were designed with BatchPrimer3 v1.0^55^ in the 200 bp flanking regions of each Y-STRs using the “SSR screening and primers” options and default parameters. To assess the specificity of the newly designed primer we performed an *in-silico* PCR using the primersearch command from EMBOSS v6.5.7.0 over all *P. anubis* genome allowing for maximum 10% of mismatches. Finally, to compare the results with human Y-STRs primers, we performed the same *in-silico* PCR on the 17 primers pairs designed in Ref.^40^ over the hg38 *H. sapiens* assembly.

### Supplementary Information

Supplementary Table S1.

Supplementary Table S2.

Supplementary Table S3.

Supplementary Table S4.

Supplementary Figures.

## Data Availability

The Y chromosome data for the Namibian *P. ursinus* sample and the two *P. papio* individuals is uploaded as mapped BAM files into the figshare repository ( https://doi.org/10.6084/m9.figshare.23810817.v2). The TYpeSTeR tool and detailed instructions on how to run it and install dependencies are available in the github repository (https://github.com/giacomomutti/TYpeSTeR).
