# Supplementary material for: Assessing the recovery of Y chromosome microsatellites with population genomic data using Papio and Theropithecus genomes
Source: Sci Rep. 2023 Aug 24;13:13839. doi: 10.1038/s41598-023-40931-x (PMC10449864; doi:10.1038/s41598-023-40931-x)
Supplement: Supplementary file 5 — Supplementary Figures. [file 41598_2023_40931_MOESM5_ESM.pdf]

Supplemental Information for:

Assessing the recovery of Y chromosome microsatellites with population genomic data using Papio and Theropithecus genomes

Table of Contents:

**Supplementary figure 1**	**1**
**Supplementary figure 2**	**2**
**Supplementary figure 3**	**3**
**Supplementary figure 4**	**4**
**Supplementary figure 5**	**4**
**Supplementary figure 6**	**5**
**Supplementary figure 7**	**6**
**Supplementary tables**	**7**

Supplementary figure 1

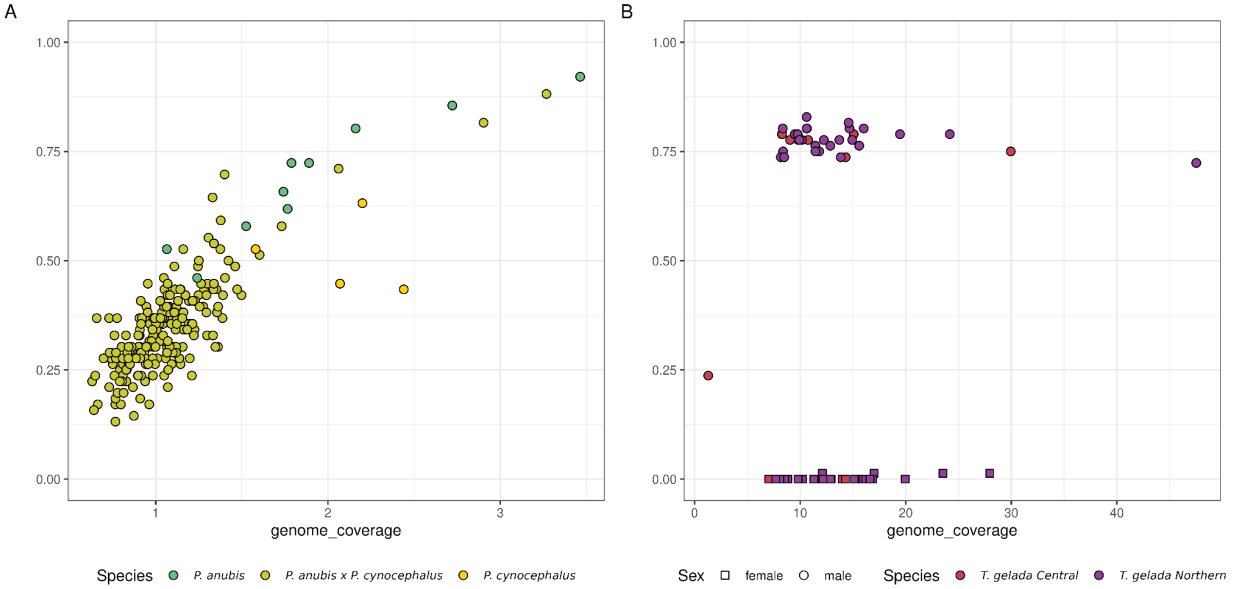

Figure S1*: Proportion of successful genotypes against whole genome sequencing coverage of each sample, coloured by species and shaped by sex of low coverage samples from Vilgalys et al, 2022 and Wall et al, 2016 (panel A) and T. gelada samples from Chiou et al, 2022 (panel B).*

Supplementary figure 2

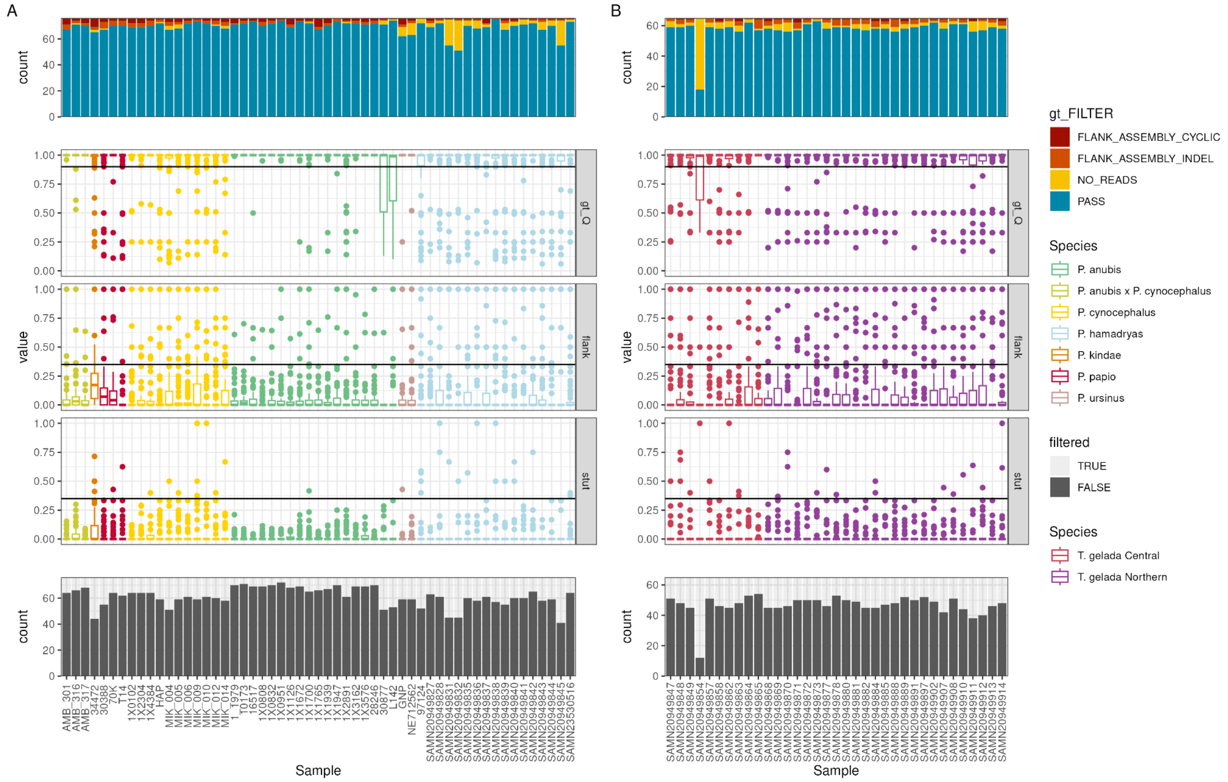

Figure S2: *Quality control of the Papio screening Y-STRs genotypes for A) Papio and B) Theropithecus samples. Each column represents an individual. Starting from the top, panels are as follows: bar plot of filters for each of the 76 tested sites colour-coded as in legend, boxplot of genotype quality, proportion of reads with an indel in the flanking regions and reads with a putative stutter artifact and finally bar plot of the number of retained or filtered genotypes.*

Supplementary figure 3

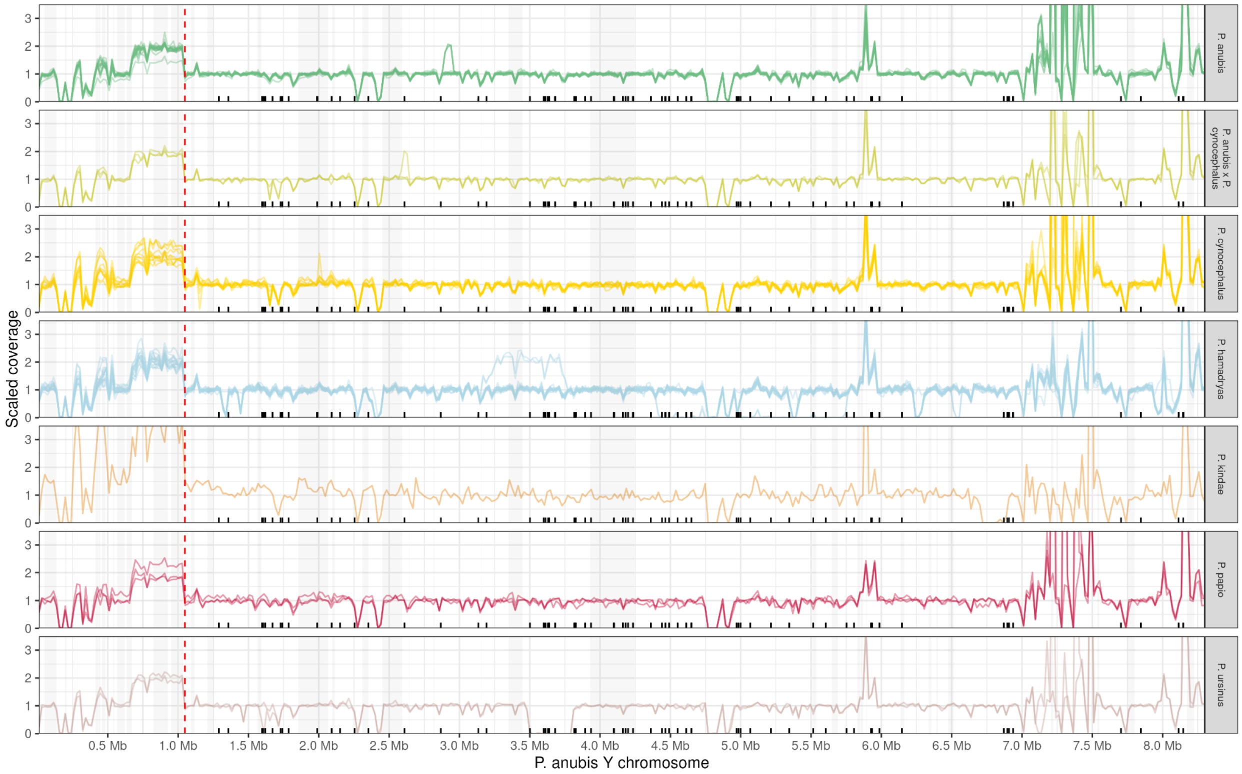

Figure S3*: Distribution of normalized coverage along the Y chromosome in samples from the Papio screening dataset, grouped according to species; Reads were mapped on the baboon reference Panubis1.0 Y chromosome (NC_044997.1). Black ticks represent the position of the 66 filtered Y-STRs and shaded grey panels represent the genes position. The Pseudo Autosomal region boundary is annotated with a red line.*

Supplementary figure 4

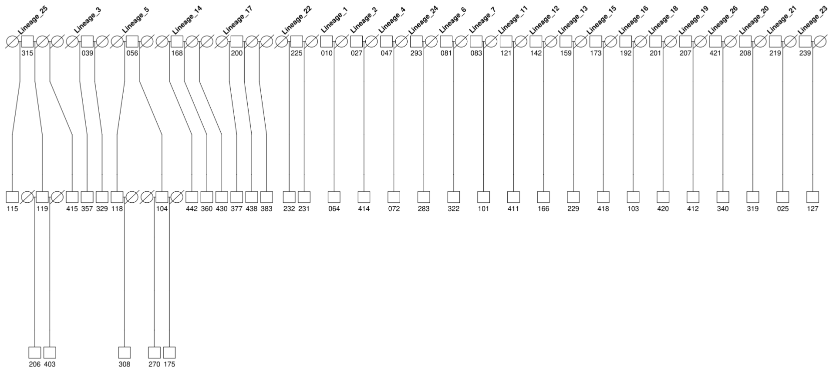

Figure S4*: Figure S4. Relationships among male Individuals in the LowCov dataset as recovered from pedigrees reported at https://github.com/TaurVil/VilgalysFogel_Amboseli_admixture (commit id: d5345cd). Lineages id and samples ids are the same as in Figure3 (Ids without "AMB_")*

Supplementary figure 5

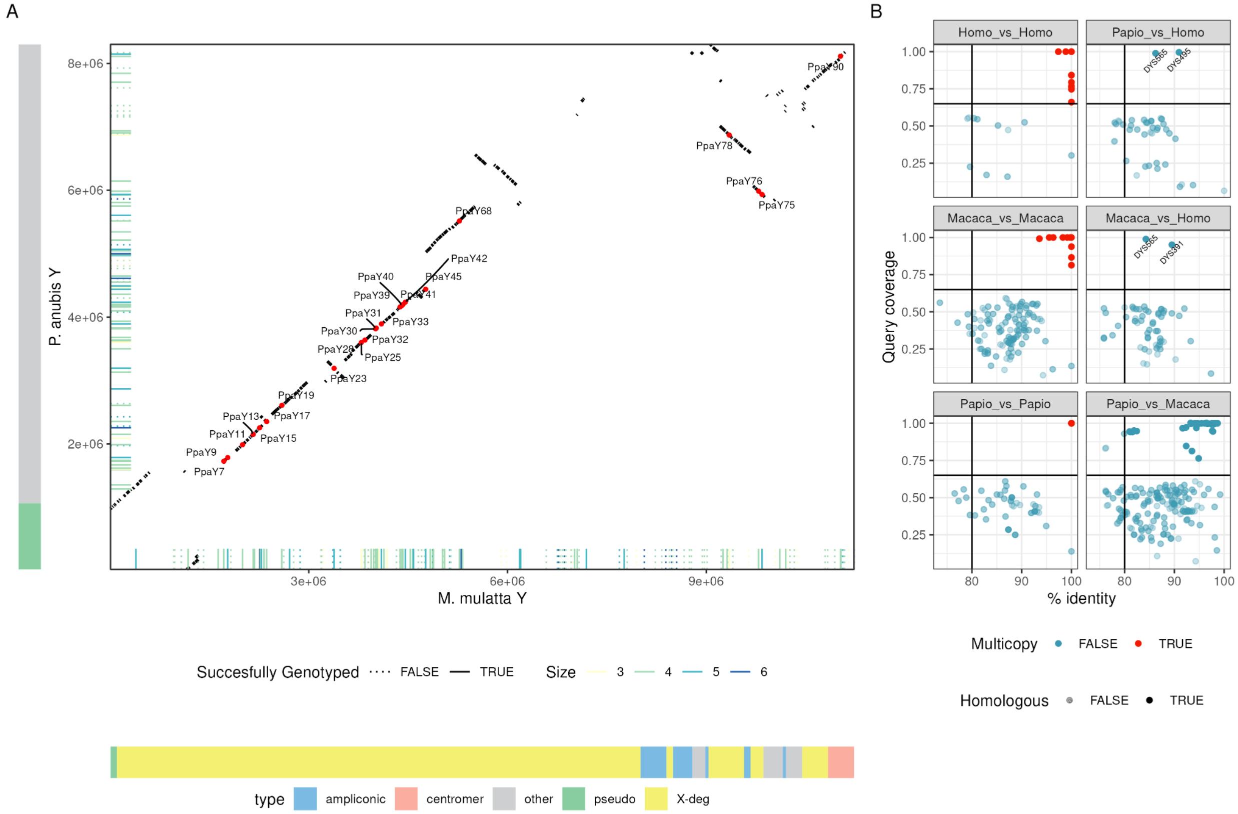

Figure S5: *A) Position of homologous (and successfully genotyped in both Papio screening dataset and Macaca dataset) Y-STRs on the Y chromosome of M. mulatta (X axis) and P. anubis (Y axis). Homologous regions were found with nucmer and are annotated with black blocks. Lines are shown for each of the 345 markers found in Macaca and the 292 found in Papio (lines are dashed if the site was not genotyped). Furthermore, for each reference chromosome the Pseudo Autosomal Regions and other chromosomal features are shown. M. mulatta regions are as described in Hughes et al. 2012. B) Percentage identity and query cover of each of the blast hits of all the Y-STRs found in Papio, Macaca and human divided by comparison. Multicopy sites are coloured in red, while hits not identified as homologous are semi transparent. A hit was filtered out if characterized by less than 80\percentage_sign identity and 0.65 query cover (thresholds annotated as black lines in the plots).*

Supplementary figure 6

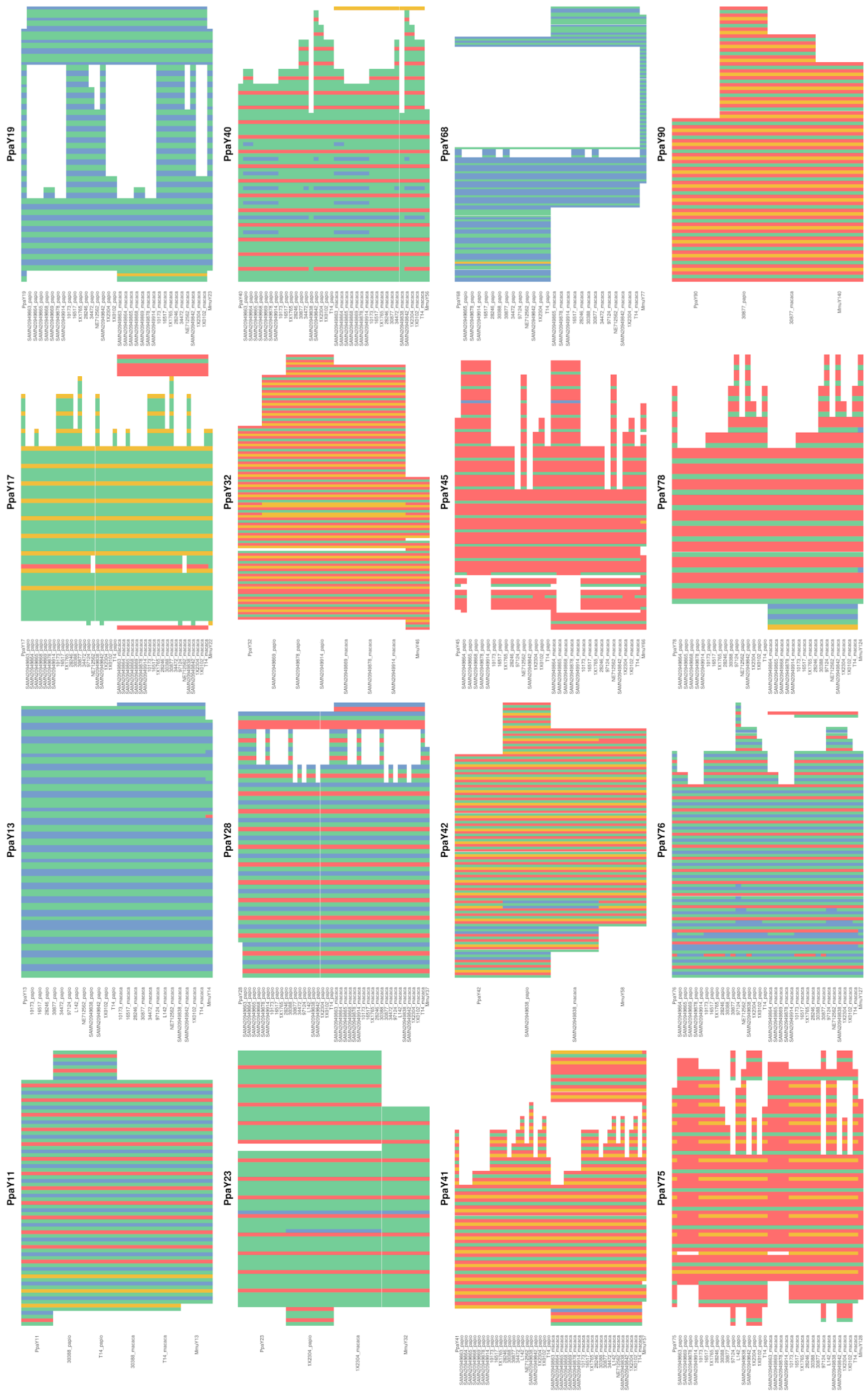

Figure S6: *Manually curated alignments of the 16 homologous markers with a different genotype between Macaca and Papio. Nucleotides are colour-coded as follows: A-red, G-yellow, T-green, C-blue and gaps are represented by white tiles. The first row is the Papio anubis region given as input to HipSTR whereas the last row is the Macaca region*

Supplementary figure 7

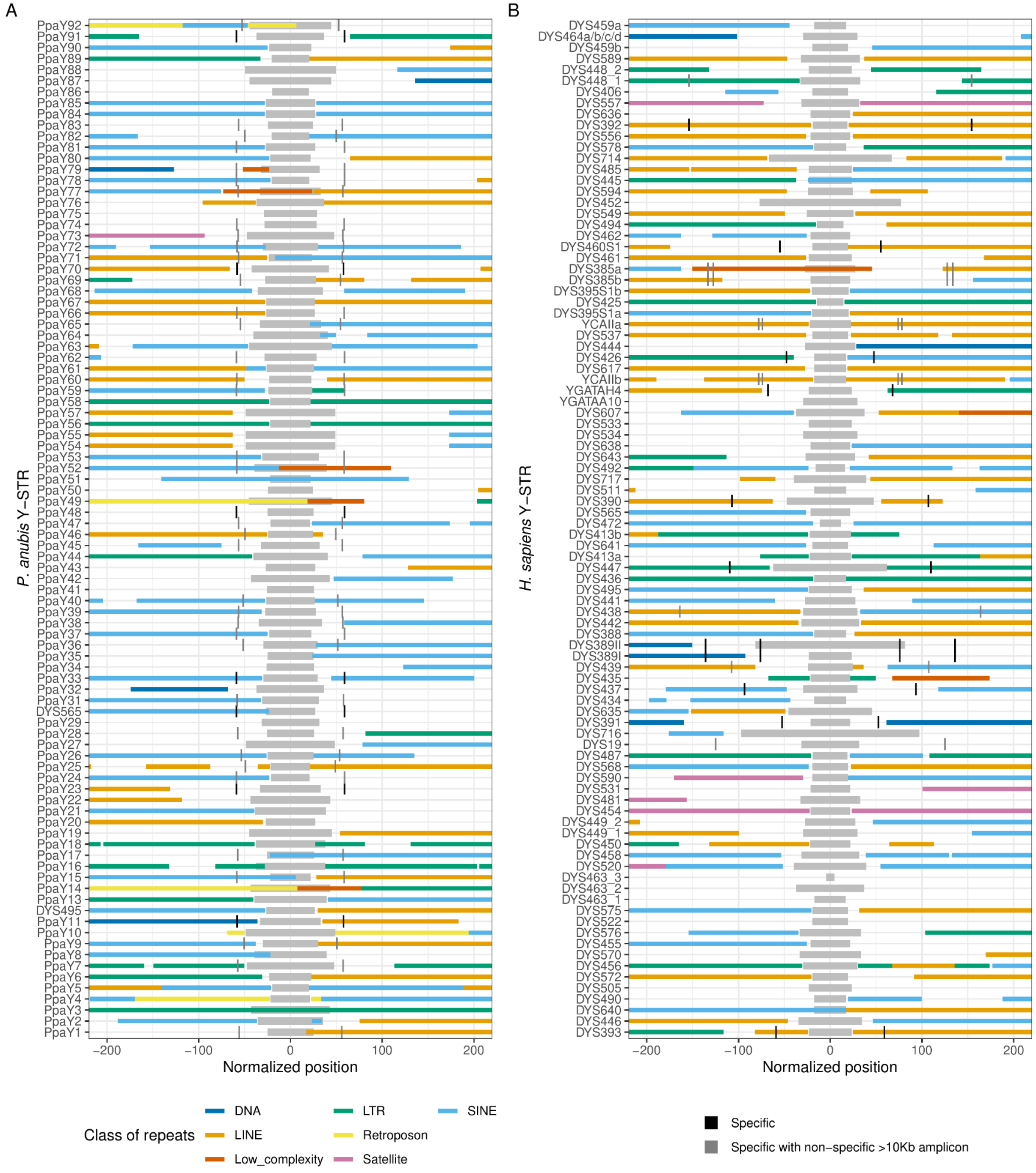

***Figure S7**: Repeated regions contained in the flanking 200bp of the 92 Papio Y-STRs (A) and the 92 "named" Y-STRs from Y-LineageTracker (B). Each STR is annotated with a grey box and the repeats are color coded by class. The primers are represented by small vertical lines and they are colored if they are truly specific or if they amplify amplicons longer than 10Kb. Positions are normalized so that the middle of the STR is zero.*

Supplementary tables

- Table S1: Information on the analysed samples. For each sample we report: the BioSample NCBI ID (when available; if the sample was sequenced with a single run we report the SRA ID), the sample name, the species, the sex, the location of origin (when known), the mean genome coverage, the source of the data ("project") and the dataset(s) it was included in this investigation. Samples identified as hybrids are reported as "*Species1xSpecies2*", referring to the two source species. For the gelada samples the annotation "Northern" and "Central" refers to the subpopulation of origin. NA: not available.
- Table S2: Final table of identified Y-STR markers. For each locus we report: start and end coordinates referring to the Y chromosome reference sequence indicated in column "reference_id", name of the locus considering the selected reference species (see Material & Methods), if multicopy or not (TRUE, FALSE), motif size and sequence, human homologous (if present), *Papio* or *Macaca* homologous (if present), reference species and Y chromosome RefSeq ID, final name considering homology (see Material & Methods), upstream flanking sequence (200bp), motif sequence, downstream flanking sequence (200bp).
- Table S3: Y-STR genotypes of the four analysed datasets (*Papio screening: A-B; Theropithecus: C-D; Macaca: E-F; LowCov: G-H;* see Materials & Methods). Each dataset has two tables: one with the filtered genotypes and one with the length of the corresponding allele. NA refers to "locus not genotyped" or "locus filtered".
- Table S4: Candidate primers for 44 *Papio* Y-STRs. For each locus are reported forward and reverse primer sequence, amplicon length (based on *P. anubis*) and Y specificity and if potential non specific fragments are longer than 10kb.
